# Atraumatic Bilateral Calcaneal Insufficiency Fractures in a Postmenopausal Woman With Severe Osteoporosis: A Case Report

**DOI:** 10.7759/cureus.116016

**Published:** 2026-09-09

**Authors:** Lung-Hsuan Lin, Chih-Wei Chang, Yu-Jou Hung

**Affiliations:** 1 Orthopedics, Education and Training Center, National Taiwan University Hospital, Taipei, TWN; 2 Orthopedics, College of Medicine, National Cheng Kung University/National Cheng Kung University Hospital, Tainan, TWN; 3 Orthopedics, Kuo General Hospital, Tainan, TWN; 4 Orthopedics, Joint Reconstruction Center, National Cheng Kung University Hospital, Tainan, TWN

**Keywords:** calcaneal fracture, heel pain, insufficiency fracture, osteoporosis, romosozumab

## Abstract

Calcaneal insufficiency fractures are uncommon and often overlooked because their early symptoms resemble more prevalent causes of heel pain. A 68-year-old postmenopausal woman presented with two months of progressive bilateral heel pain and swelling without trauma. Radiographs revealed nondisplaced bilateral calcaneal fractures characterized by subtle vertical lucencies and mild cortical discontinuity. Dual-energy X-ray absorptiometry confirmed severe osteoporosis, and laboratory evaluation excluded secondary metabolic bone disease. The initial symptoms mimicked plantar fasciitis and Achilles tendinopathy, contributing to diagnostic delay. Conservative management, including protected weight bearing, romosozumab, calcium, and vitamin D supplementation, resulted in steady improvement, with pain-free ambulation achieved within three months. Follow-up radiographs demonstrated complete resolution of the fracture line and restoration of normal trabecular architecture. Persistent atraumatic heel pain in elderly patients should prompt evaluation for calcaneal insufficiency fractures, and early imaging combined with bone mineral density assessment is essential for timely diagnosis and optimal management.

## Introduction

Bilateral calcaneal fractures are typically associated with high-energy trauma, such as falls from height [1,2]. In contrast, stress fractures can be categorized into fatigue fractures, resulting from abnormal load on normal bone, and insufficiency fractures, caused by normal physiological loading on structurally weakened bone, most commonly due to osteoporosis or metabolic bone disorders [3,4]. Compared with insufficiency fractures of the tibia or femur, calcaneal insufficiency fractures are less frequently reported, and bilateral involvement is particularly rare [3]. Early symptoms often mimic plantar fasciitis, Achilles tendinopathy, or nonspecific heel pain, leading to potential diagnostic delay. In elderly individuals with severe osteoporosis, persistent atraumatic heel pain should prompt careful evaluation for insufficiency fractures. This report describes a rare case of bilateral calcaneal insufficiency fractures in a woman with severe osteoporosis and highlights the importance of early recognition.

## Case presentation

A 68-year-old woman with a BMI of 21.4 kg/m² presented with two months of insidious bilateral heel pain and swelling without trauma or increased activity. Her medical history was unremarkable, with no renal, endocrine, or metabolic abnormalities. On inspection, the patient demonstrated mild pes planus with neutral hindfoot alignment and no visible plantar fat-pad atrophy, providing no anatomical explanation for bilateral symptoms.

Physical examination revealed bilateral heel tenderness with mild soft tissue swelling, and neurovascular status was intact. Focal tenderness was elicited over both the medial and lateral calcaneal walls, and a mediolateral calcaneal squeeze test yielded bilateral positive results. Under the initial impression of inadequately treated plantar fasciitis, radiographs were obtained. Instead of traction spurs or plantar soft tissue changes, the images demonstrated nondisplaced bilateral calcaneal fractures without subtalar joint involvement. Given the absence of trauma and the osteopenic appearance, these findings were consistent with insufficiency rather than fatigue fractures. Subtle vertical lucent lines over the posterior calcaneal body, mild cortical discontinuity, and diffuse osteopenia were noted (Figure 1).

**Figure 1:**
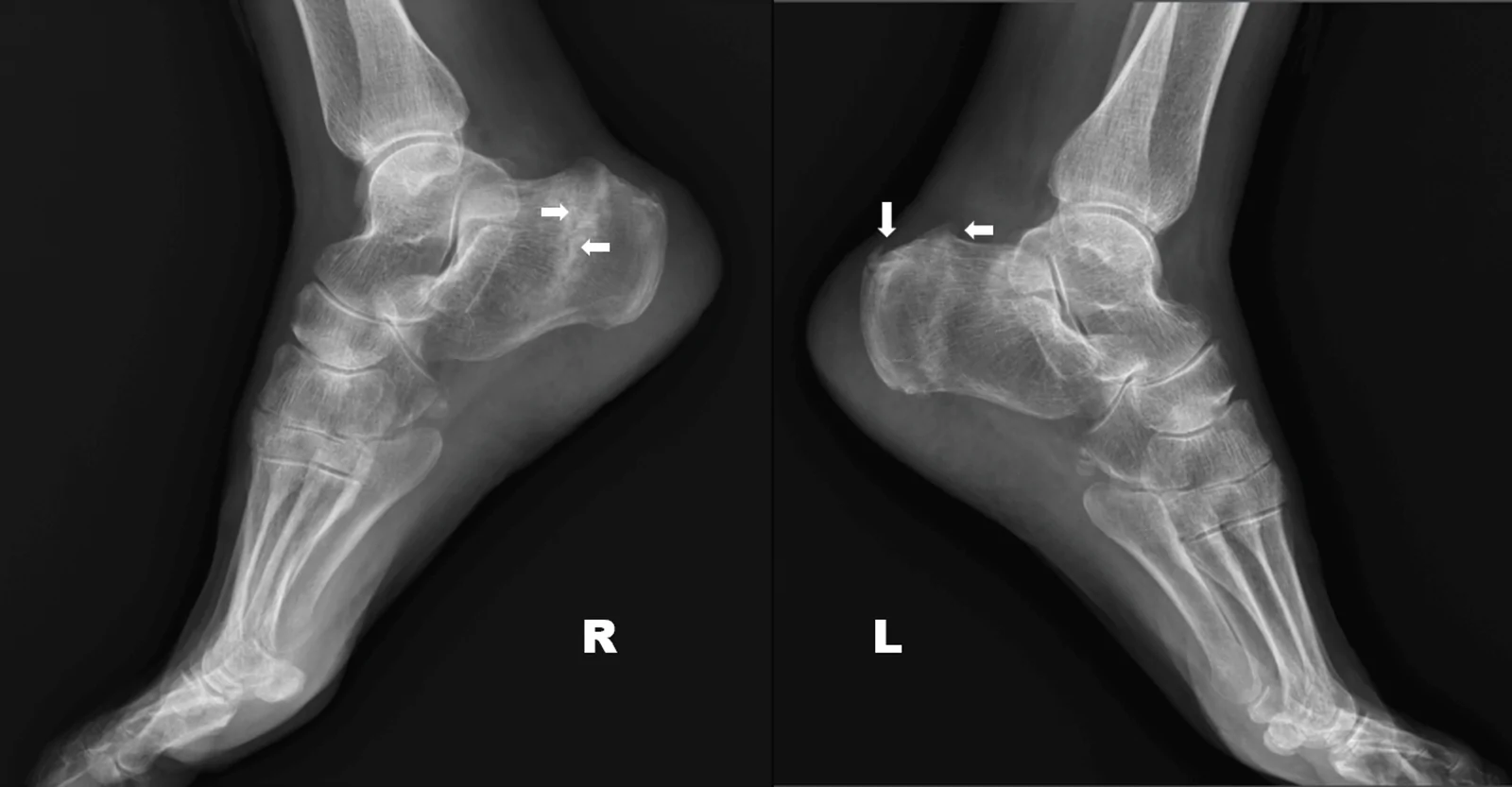
Initial lateral radiographs of the calcaneus The initial radiographs demonstrate nondisplaced bilateral calcaneal insufficiency fractures. In the right calcaneus, the arrowheads indicate subtle vertical radiolucent lines within the posterior calcaneal body. In the left calcaneus, the arrowheads highlight mild cortical discontinuities along the posterior calcaneal cortex. Both findings are consistent with early insufficiency fractures in the setting of severe osteoporosis.

Given the osteopenic appearance, further evaluation was performed. Dual-energy X-ray absorptiometry (DXA) confirmed severe osteoporosis, with lumbar spine L1-L4 bone mineral density (BMD) 0.674 g/cm² (T-score -3.6) and femoral neck BMD 0.597 g/cm² (T-score -3.4) (Figure 2). Laboratory studies, including renal function (blood urea nitrogen (BUN) 23 mg/dL, range 8-23; creatinine 0.85 mg/dL, range 0.51-0.95), calcium (8.9 mg/dL, range 8.6-10), phosphate (4.2 mg/dL, range 2.5-4.5), parathyroid hormone (31.8 pg/mL, range 15-65), and thyroid function (T3, T4, thyroid-stimulating hormone (TSH)), were normal, excluding secondary metabolic bone disease.

**Figure 2:**
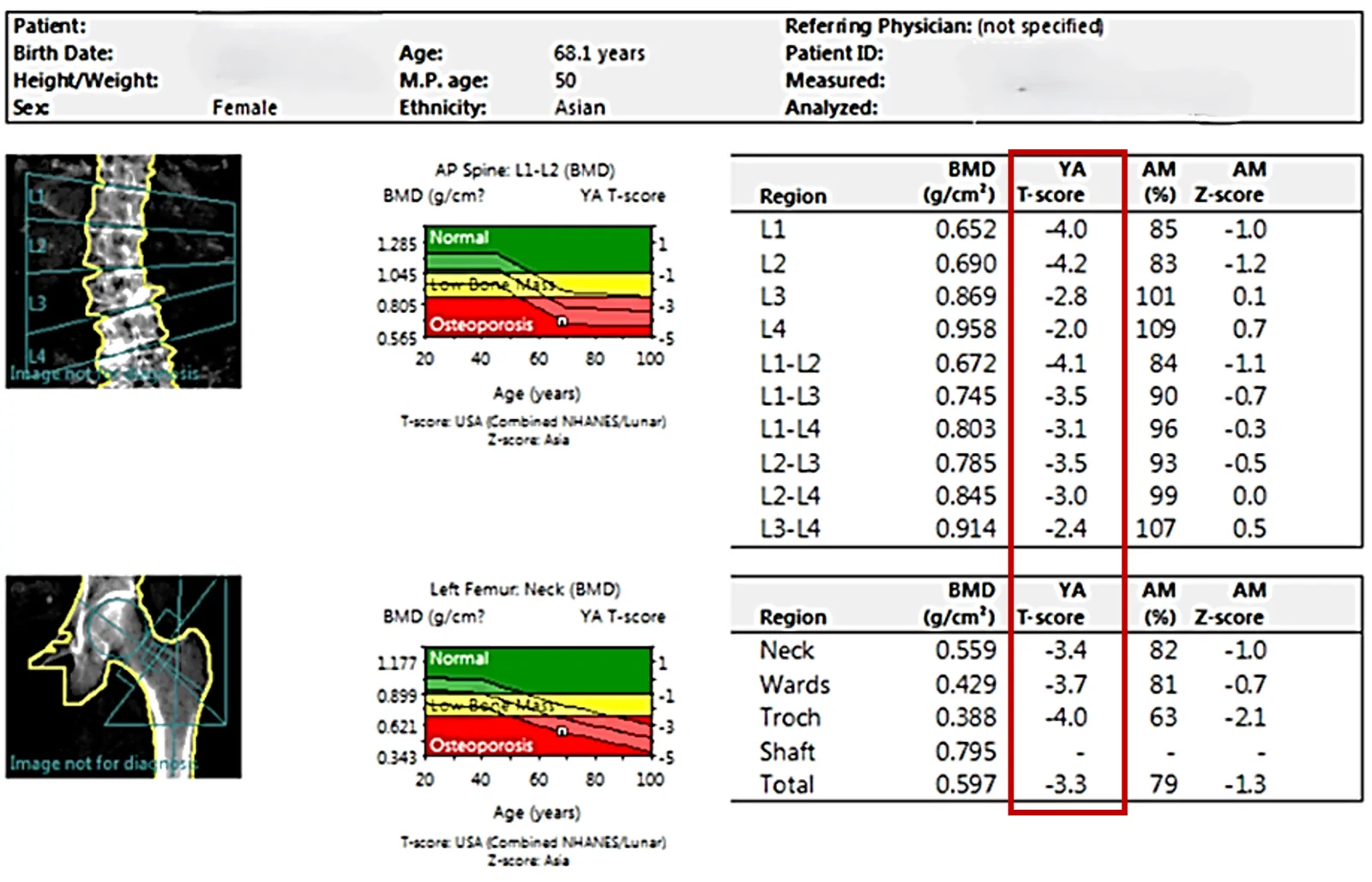
DXA demonstrating severe osteoporosis DXA demonstrates severe osteoporosis, with markedly reduced BMD at the lumbar spine (L1-L4 BMD 0.674 g/cm², T‑score -3.6) and the left femoral neck (BMD 0.597 g/cm², T‑score -3.4). Regions of interest for the spine and proximal femur are outlined for quantitative analysis; the DXA raw image contains OCR artifacts; quantitative values were manually verified. BMD, bone mineral density; DXA, dual-energy X-ray absorptiometry; OCR, optical character recognition

Protected weight bearing, monthly romosozumab injections, and calcium/vitamin D supplementation were initiated. Symptoms improved steadily, and she regained pain-free ambulation within three months. Follow-up radiographs demonstrated disappearance of the fracture line and restoration of normal trabecular architecture (Figure 3).

**Figure 3:**
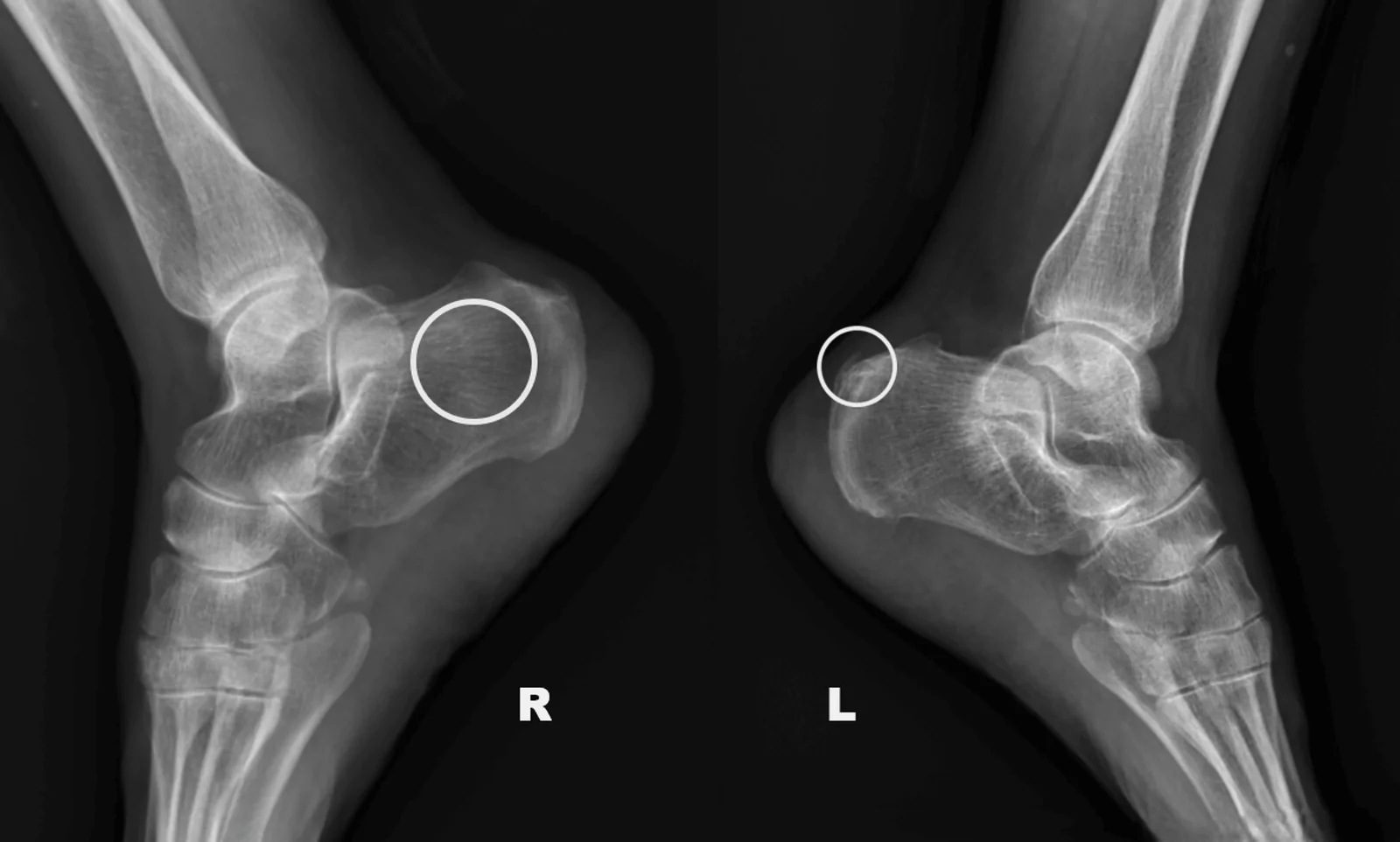
Follow-up lateral radiographs of the calcaneus The follow-up radiographs demonstrate interval healing of bilateral calcaneal insufficiency fractures. The circled regions indicate trabecular reconstitution and progressive fracture consolidation within the posterior calcaneal body, corresponding to resolution of the previously noted vertical radiolucent line and cortical discontinuity.

## Discussion

Calcaneal insufficiency fractures are uncommon and may be overlooked because of nonspecific symptoms and subtle early radiographic findings [3]. Bilateral involvement is particularly rare, with only a few cases reported [5]. In this patient, the absence of trauma, the presence of severe osteoporosis, and the bilateral distribution strongly support an insufficiency mechanism rather than a fatigue fracture. Fatigue fractures typically occur in younger or physically active individuals subjected to repetitive high-impact loading, whereas insufficiency fractures arise when normal physiological stress is applied to weakened bone. Collectively, these clinical and radiographic features are consistent with the characteristic pattern of insufficiency fractures described in prior literature.

The early clinical presentation resembled plantar fasciitis or Achilles tendinopathy, which are far more common causes of heel pain. Similar diagnostic challenges have been reported, with patients initially treated for soft-tissue disorders before imaging revealed insufficiency fractures [3,6]. Pierson et al. [7] also emphasized frequent misdiagnosis due to overlapping symptoms and subtle radiographic findings. Unlike their patient, who required surgical intervention for nonunion, our patient responded well to conservative management. In this case, focal tenderness over the medial and lateral calcaneal walls and a positive mediolateral squeeze test helped distinguish calcaneal fractures from plantar fasciitis, directly supporting the diagnostic distinctions summarized in Table 1. Additionally, the patient’s mild pes planus, neutral hindfoot alignment, and preserved plantar fat-pad thickness excluded anatomical contributors to bilateral symptoms.

**Table 1:** Clinical and imaging differentiation among plantar fasciitis, calcaneal insufficiency fracture, and calcaneal fatigue fracture Source [3,5-8,10]

Feature	Plantar Fasciitis	Calcaneal Insufficiency Fracture	Calcaneal Fatigue Fracture
Typical patient profile	Middle‑aged; overweight	Elderly; osteoporotic	Young; athletes; military recruits
Mechanism	Traction injury	Normal load on weakened bone	Repetitive high‑impact loading
Pain characteristics	Plantar medial heel; worst with first steps	Posterior calcaneal body; worsens with weight-bearing	Posterior calcaneus; worsens with activity
Exam findings	Positive windlass test; no swelling; negative mediolateral squeeze test	Focal bony tenderness; mild swelling; positive mediolateral squeeze test	Focal bony tenderness; possible swelling; positive mediolateral squeeze test
Imaging features	Radiographs often normal; fascia thickening on MRI	Vertical lucency/cortical discontinuity; fracture line with marrow edema; MRI more sensitive for early detection	Radiographs often normal; fracture line with periosteal reaction; MRI shows early marrow edema
Distinguishing factors	Improves with soft‑tissue treatment; normal bone quality	No improvement with soft‑tissue treatment; osteopenic bone	No improvement with soft‑tissue treatment; normal bone quality

Differentiating plantar fasciitis, calcaneal insufficiency fractures, and calcaneal fatigue fractures is clinically important because early symptoms overlap substantially. Table 1 summarizes key distinctions in clinical presentation and imaging characteristics among these entities.

Previous reports have described unilateral insufficiency fractures associated with osteoporosis, rheumatoid arthritis, chronic steroid use, or anticonvulsant therapy [3-6,8,9]. Bilateral cases are even less common, as illustrated by the reports of Kaya et al. and Hirose et al. Kaya et al. linked chronic carbamazepine use to bilateral calcaneal insufficiency fractures [10], while Hirose et al. recently described a patient with rheumatoid arthritis in whom bilateral calcaneal insufficiency fractures were initially mistaken for inflammatory heel pain until MRI revealed bilateral fracture lines [11]. In contrast, our patient had no secondary metabolic bone disease, chronic inflammatory disorder, or medication exposure, suggesting that severe primary osteoporosis alone was sufficient to precipitate bilateral insufficiency fractures.

Romosozumab was administered as part of osteoporosis management. Although anabolic agents can enhance bone formation, their effect on fracture healing remains uncertain, and no causal relationship can be inferred from a single case. Existing evidence shows that romosozumab increases bone formation and improves bone strength in clinical trials evaluating fracture-risk reduction [12,13], but these studies were not designed to assess healing dynamics. In this patient, strict adherence to protected weight bearing was likely the primary biomechanical driver of short-term clinical union at three months, while romosozumab addressed the profound systemic deficit (T-score -3.6) to reduce future fracture risk rather than directly accelerating fracture healing. This clarification tempers the interpretation of the temporal association between romosozumab initiation and radiographic improvement.

Limitations of this report include the single-patient design, lack of advanced imaging such as MRI or CT, and inability to isolate the effect of romosozumab. MRI was not performed because the radiographic findings, combined with DXA-confirmed severe osteoporosis, were considered sufficiently diagnostic, and the patient did not meet reimbursement criteria for advanced imaging in the absence of trauma. Despite these limitations, this case reinforces the importance of considering insufficiency fractures in elderly patients with unexplained heel pain. Early recognition and appropriate management, including protected weight bearing and osteoporosis treatment, may prevent fracture progression and improve clinical outcomes.

## Conclusions

Bilateral calcaneal insufficiency fractures are uncommon and often present with nonspecific atraumatic heel pain, making early diagnosis challenging. In elderly patients, persistent heel pain should prompt evaluation for stress-related bony injuries, with BMD assessment guiding interpretation and management. Early recognition and appropriate management, including protected weight-bearing combined with systemic osteoporosis therapy, may prevent fracture progression and optimize functional recovery.
